# The relationship between HbA1c values and the occurrence of hypoglycemia as assessed by continuous glucose monitoring in patients with type 1 diabetes

**DOI:** 10.1186/s13098-016-0167-z

**Published:** 2016-07-29

**Authors:** Daisuke Tsujino, Rimei Nishimura, Yoshiko Onda, Chiaki Seo, Kiyotaka Ando, Aya Morimoto, Kazunori Utsunomiya

**Affiliations:** 1 Division of Diabetes, Metabolism and Endocrinology, Department of Internal Medicine, Jikei University School of Medicine, 3-15-8 Nishishinbashi, Minato-ku, Tokyo, 105-8461 Japan; 2 Graduate School of Public Health, University of Pittsburgh, Pittsburgh, PA USA; 3 Morimoto Hospital, Tokyo, Japan

**Keywords:** Continuous glucose monitoring, CGM, Type 1 diabetes, HbA1c, Hypoglycemia

## Abstract

**Background:**

We aimed to examine the relationship between the occurrence of hypo-/hyperglycemia and HbA1c values, as assessed by continuous glucose monitoring (CGM) in patients with type 1 diabetes.

**Methods:**

The study subjects comprised 101 type 1 diabetic patients on basal-bolus insulin therapy, who were put on masked CGM immediately after admission. The subjects were divided into four groups equally by HbA1c values and the 24-h CGM data were compared among the groups.

**Results:**

Groups A to D comprised 24 patients with HbA1c ≤7.2 %, 26 patients with 7.2 % <HbA1c ≤8.2 %, 27 patients with 8.2 % <HbA1c ≤9.2 %, and 24 patients with HbA1c >9.2 %, respectively. The higher the HbA1c values, the significantly higher the 24-h mean glucose levels [median (25–75 percentiles)], with the HbA1c in groups A to D being 133 (114–155), 158 (132–188), 182 (152–206), and 186 (143–215) mg/dL, respectively (*P* < 0.001). Conversely, the higher the HbA1c values, the significantly shorter the time in hypoglycemia (<70 mg/dL), with the time in groups A to D being 170 (58–341), 78 (0–210), 45 (0–105), and 20 (0–105) min, respectively (*P* = 0.014); and the higher the HbA1c values, the significantly shorter the time in nocturnal hypoglycemia, with the time in groups A to D being 120 (5–269), 25 (0–120), 0 (0–60), and 0 (0–89) min, respectively (*P* = 0.019). No significant difference was seen between groups A to D in the standard deviations (SDs) of 24-h glucose values at 53 (40–65), 54 (45–70), 64 (55–76), and 58 (48–80), respectively.

**Conclusion:**

In type 1 diabetic patients, lower HbA1c was not associated with lower SD of 24-h glucose values, but may result in increased hypoglycemia.

*Trial Registration* Current controlled trials UMIN000019190

## Background

Currently, the self-monitoring of blood glucose (SMBG) is widely used as a modality to help patients with diabetes keep track of their blood glucose levels on a daily basis. However, while they may appear on SMBG (i.e., a few measurements a day) to remain within the normoglycemic range, they may be shown on continuous glucose monitoring (CGM) to have otherwise unrecognized glycemic fluctuations or hypoglycemic episodes after meals or during the night. Thus, the greatest benefit of CGM is derived from its ability to detect and make visible glycemic fluctuations over a 24-h period.

In this regard, real-time CGM, which is mainly used in routine clinical practice in the US and some European countries, may be of particular interest, in that it allows glucose levels as they are measured in patients with diabetes to be immediately displayed on screen. When clues are provided as an indication of hyperglycemia, patients may need an additional injection of rapid-acting insulin or need to eat less than usual. Thus, real-time CGM appears to have great potential as an ultimate self-management tool for patients with diabetes. Indeed, real-time CGM-based intervention in type 1 diabetic patients has led to significant improvements in HbA1c in those 25 years of age or older in a large-scale clinical trial [1].

In contrast, “masked” CGM, which does not allow data to be displayed on a real-time basis, may prove more helpful than real-time CGM in that it provides an opportunity to review patterns of glycemic fluctuations occurring over a certain period, to identify factors responsible for any hypoglycemic or hyperglycemic episode observed, and to optimize treatments for each individual patient [2, 3].

Ever since the publication of the ACCORD study results, it has been commonly assumed that the occurrence of serious hypoglycemia may likely increase the risk for cardiovascular events or mortality in type 2 diabetic patients with high HbA1c values [4]. To date, however, very few studies have explored whether varying HbA1c values may account for different durations of hypoglycemia in type 2 diabetic patients receiving hypoglycemic drugs that could cause hypoglycemia. Thus, we conducted a CGM-based study to compare durations of hypoglycemia and hyperglycemia in 40 type 2 diabetic patients receiving sulfonylureas, glinides, or insulin, which demonstrated that those with high HbA1c values were associated with longer durations of hyperglycemia than those with low HbA1c, while there were no significant differences in the proportion of patients with hypoglycemia and in the duration of hypoglycemia between those with high HbA1c values and those with low HbA1c values [5].

Given that still fewer studies have addressed this issue in type 1 diabetic patients, we conducted a similar study in type 1 diabetic patients receiving basal-bolus insulin therapy to examine the relationship between their HbA1c values and their 24-h glycemic fluctuations—particularly their hypoglycemic episodes—as captured retrospectively by masked CGM.

## Methods

The study included a total of 101 patients with type 1 diabetes being treated with basal-bolus insulin therapy [insulin glargine or insulin detemir used as basal insulin, and insulin aspart or insulin lispro as bolus insulin; continuous subcutaneous insulin infusion (CSII) excluded] at our hospital, each of whom was subjected to CGM using CGMS GOLD (Medtronic, Inc. Northridge, CA, USA) immediately after admission. Written informed consent was obtained from participants of this study. In this study, care was taken to ensure that the carbohydrate content was nearly consistent for all three meals provided as the hospital-provided diet. Again, the subjects were allowed to ingest a minimum amount of glucose when they developed severe hypoglycemia. The first serially measured values from 0:00 to 24:00 were included for analysis. Mean of daily difference (MODD) were estimated with the next 24-h data. In addition, the study required that the CGM readings be calibrated by SMBG values 4 times or more during the 24-h period.

Based on the HbA1c values measured in these patients within a month of their admission, the patients were divided into four groups equally, each of which accounted for more or less the same number of patients: group A, HbA1c ≤7.2 %; group B, 7.2 % <HbA1c ≤8.2 %; group C, 8.2 % <HbA1c ≤9.2 %; and group D, HbA1c >9.2 %.

The HbA1c subgroups were compared for 24-h mean glucose values and their standard deviations (SDs), mean amplitude of glycemic excursions (MAGE),  % coefficient of variation (CV), J-index, M value, MODD, time in hypoglycemia, time in nocturnal (23:00–6:00) hypoglycemia, low blood glucose index (LBGI), time in hyperglycemia, and high blood glucose index (HBGI).

Next, the HbA1c values were examined for correlation with increases or decreases in the frequency of hypoglycemia or nocturnal hypoglycemia in these subgroups. Hypoglycemia and hyperglycemia were defined as glucose <70 and >180 mg/dL, respectively.

All values were expressed as medians (25–75 percentiles). The four HbA1c subgroups were compared by using the Kruskal–Wallis test. All statistical analyses were performed by using SPSS (version 19).

This study was conducted with the approval of the Institutional Review Board of the Jikei University School of Medicine, Tokyo, Japan.

## Results

Groups A to D accounted for a total of 24, 26, 27, and 24 patients, respectively, with a female predominance in all groups (Table 1). These subgroups were not significantly different with regard to age (*P* = 0.956), duration of diabetes (*P* = 0.653), and urinary C-peptide immunoreactivity (CPR) (*P* = 0.622), but were significantly different in body mass index (BMI) (*P* = 0.007).

**Table 1:** The patient demographic data and the dose of insulin for 101 type 1 diabetic patients on basal-bolus insulin therapy as stratified equally by HbA1c value

HbA1c	Group A HbA1c ≤7.2 %	Group B 7.2 % <HbA1c ≤8.2 %	Group C 8.2 % <HbA1c ≤9.2 %	Group D HbA1c >9.2 %	*P* value
Males /females^a^	5/19	8/18	10/17	9/15	0.518
Age (years)^b^	36.5 (32.0–58.8)	41.5 (31.5–54.5)	41.0 (32.0–55.0)	42.0 (35.0–57.8)	0.956
Duration of diabetes (years)^b^	11.0 (5.3–30.0)	15.0 (7.8–25.5)	15.0 (5.0–24.0)	10.5 (7.3–15.0)	0.653
BMI (kg/m^2^)^b^	20.3 (18.2–22.3)	22.6 (21.0–24.9)	22.4 (20.0–23.8)	22.2 (20.3–25.3)	*0.007*
U-CPR (µg/day)^b^	1.2 (0.9–3.9)	0.9 (0.6–2.6)	1.3 (0.7–3.6)	1.0 (0.5–6.0)	0.622
Total daily dose of insulin (units)^b^	31.0 (25.5–38.8)	35.0 (26.5–41.5)	37.0 (29.0–46.0)	39.0 (31.3–51.5)	0.099
Basal insulin dose (units)^b^	13.5 (9.3–19.5)	14.0 (10.0–20.3)	15.0 (12.0–18.0)	18.5 (14.0–24.8)	*0.021*
Bolus insulin dose (units)^b^	17.0 (15.0–24.0)	19.5 (15.0–24.3)	20.0 (16.0–30.0)	21.5 (13.8–27.5)	0.296

Data presented as median (25–75 percentiles)

Italic values indicate significance of *P* value (*P* < 0.05)

^a^Chi square test

^b^Kruskal–Wallis test

The subgroups were not significantly different with regard to the total daily dose of insulin (TDD) (*P* = 0.099). Of the TDD, the basal insulin dose was significantly different between the subgroups (*P* = 0.021) and the higher the HbA1c values, the more the basal insulin dose. However, the bolus insulin dose was not significant (*P* = 0.296).

Table 2 summarizes 24-h CGM data. A comparison of the HbA1c subgroups showed that the higher the HbA1c, the significantly higher the mean glucose values (P < 0.001); and, conversely, that the higher the HbA1c, the significantly shorter the time in hypoglycemia (P = 0.014) and the time in nocturnal hypoglycemia (P = 0.019); while, interestingly, the SDs of 24-h glucose values, MAGE, %CV, M values, and MODD were not significantly different between the subgroups.

**Table 2:** 24-h CGM data of each HbA1c group

HbA1c	Group A HbA1c ≤7.2 %	Group B 7.2 % <HbA1c ≤8.2 %	Group C	Group D HbA1c >9.2 %	*P* value
			8.2 % <HbA1c ≤9.2 %		
Mean glucose levels (mg/dL)^a^	133.0 (113.5‒154.8)	157.5 (131.5‒187.5)	182.0 (152.0‒206.0)	186.0 (143.0‒214.8)	*<0.001*
SD^a^	53.0 (40.0‒65.0)	53.5 (44.8‒69.8)	64.0 (55.0‒76.0)	58.0 (48.0‒80.0)	0.165
*MAGE* (*mg/dL*)^*a*^	*99.3* (*62.4–149.0*)	*99.7* (*78.5–130.3*)	*103.3* (*81.8–127.9*)	*89.8* (*66.7–119.6*)	0.783
*%CV* ^*a*^	*40.7* (*32.8–51.2*)	*34.0* (*30.8–45.1*)	*37.6* (*30.1–41.0*)	*36.8* (*27.1–45.6*)	0.405
*J-index* ^*a*^	*38.7* (*24.8–44.1*)	*51.0* (*31.5–61.5*)	*64.7* (*44.1–82.6*)	*65.2* (*40.5–82.8*)	*<0.001*
*M value* ^*a*^	*10.9* (*6.7–20.2*)	*15.1* (*7.6–18.3*)	*17.5* (*10.3–29.9*)	*19.4* (*9.6–27.4*)	0.126
*MODD* (*mg/dL*)^*a*^	*43.9* (*31.3–54.4*)	*53.5* (*41.8–64.1*)	*53.5* (*39.7–89.6*)	*57.7* (*41.0–81.4*)	0.163
Time in hypoglycemia (min)^a^	170.0 (57.5‒341.3)	77.5 (0‒210.0)	45.0 (0‒105.0)	20.0 (0‒105.0)	*0.014*
Time in nocturnal hypoglycemia (min)^a^	120.0 (5.0‒268.8)	25.0 (0‒120.0)	0 (0‒60.0)	0 (0‒88.8)	*0.019*
*LBGI* ^*a*^	*5.7* (*2.6–9.0*)	*3.6* (*0.9–6.8*)	*3.9* (*1.2–10.8*)	*2.2* (*0.6–5.6*)	0.162
Time in hyperglycemia (min)^a^	335.0 (147.5‒446.3)	457.5 (193.8‒722.5)	715.0 (425.0‒945.0)	675.0 (351.3‒953.8)	*0.001*
*HBGI* ^*a*^	*8.1* (*5.4–9.3*)	*10.9* (*6.0–13.8*)	*13.2* (*8.4–19.4*)	*13.7* (*6.6–20.4*)	*0.003*

Data presented as median (25–75 percentiles)

Italic values indicate significance of *P* value (*P* < 0.05)

^a^Kruskal–Wallis test

Table 3 summarizes the indices for glycemic variability for every 6-h segment, i.e., 0 a.m.–6 a.m., 6 a.m.–12 noon, 12 noon–6 p.m., and 6 p.m.–12 midnight, over 24 h as compared between all HbA1c subgroups. While mean glucose levels were significantly different between the HbA1c subgroups except for the 6 a.m.–12 noon segment, SD and %CV were not significantly different between the subgroups.

**Table 3:** Indices for glycemic variability for every 6-h segment over 24 h in all HbA1c subgroups

HbA1c	Group A HbA1c ≤7.2 %	Group B 7.2 % <HbA1c ≤8.2 %	Group C 8.2 % <HbA1c ≤9.2 %	Group D HbA1c >9.2 %	*P* value
0 a.m.–6 a.m. Mean glucose levels (mg/dL)	86.5 (66.9–117.2)	99.8 (85.6–144.1)	135.0 (82.0–165.8)	127.2 (84.2–197.4)	*0.020*
6 a.m.–12 noon Mean glucose levels (mg/dL)	158.3 (132.8–208.6)	176.3 (145.5–224.1)	188.5 (153.5–251.1)	174.5 (143.7–237.1)	0.105
12 noon–18 p.m. Mean glucose levels (mg/dL)	127.3 (108.9–149.6)	133.3 (120.6–190.9)	190.5 (146.4–247.8)	177.4 (120.7–250.3)	*0.007*
18 p.m.–12 midnight Mean glucose levels (mg/dL)	156.8 (110.1–195.8)	161.5 (126.1–234.8)	194.2 (150.1–245.2)	210.2 (139.3–234.9)	*0.013*
0 a.m.–6 a.m. SD (mg/dL)	19.6 (10.8–25.3)	18.2 (11.5–30.2)	20.6 (13.4–31.8)	23.0 (13.3–36.4)	0.402
6 a.m.–12 noon SD (mg/dL)	37.7 (28.4–59.2)	40.2 (32.7–63.9)	40.5 (35.8–68.0)	47.1 (30.7–57.2)	0.604
12 noon–18 p.m. SD (mg/dL)	29.6 (18.1–41.2)	27.7 (18.8–41.7)	30.4 (21.6–51.7)	33.5 (26.6–48.0)	0.446
18 p.m.–12 midnight SD (mg/dL)	35.5 (22.5–44.9)	33.8 (28.3–51.5)	35.6 (24.3–43.6)	36.7 (25.2–54.1)	0.891
0 a.m.–6 a.m. % CV	22.6 (14.1–33.3)	19.5 (10.6–25.1)	17.9 (12.8–27.3)	17.2 (11.8–26.9)	0.608
6 a.m.–12 noon % CV	26.3 (15.2–35.7)	23.7 (18.3–30.9)	23.5 (18.5–30.7)	25.0 (17.6–33.5)	0.978
12 noon–18 p.m. % CV	17.9 (14.6–35.7)	20.0 (16.1–27.0)	18.1 (11.1–28.2)	19.9 (13.3–29.6)	0.905
18 p.m.–12 midnight % CV	23.6 (16.9–28.0)	21.9 (17.3–27.4)	17.8 (12.2–23.2)	21.0 (11.5–25.3)	0.136

Data presented as median (25–75 percentiles)

Italic values indicate significance of *P* value (*P* < 0.05)

^a^Kruskal–Wallis test

**Fig. 1:**
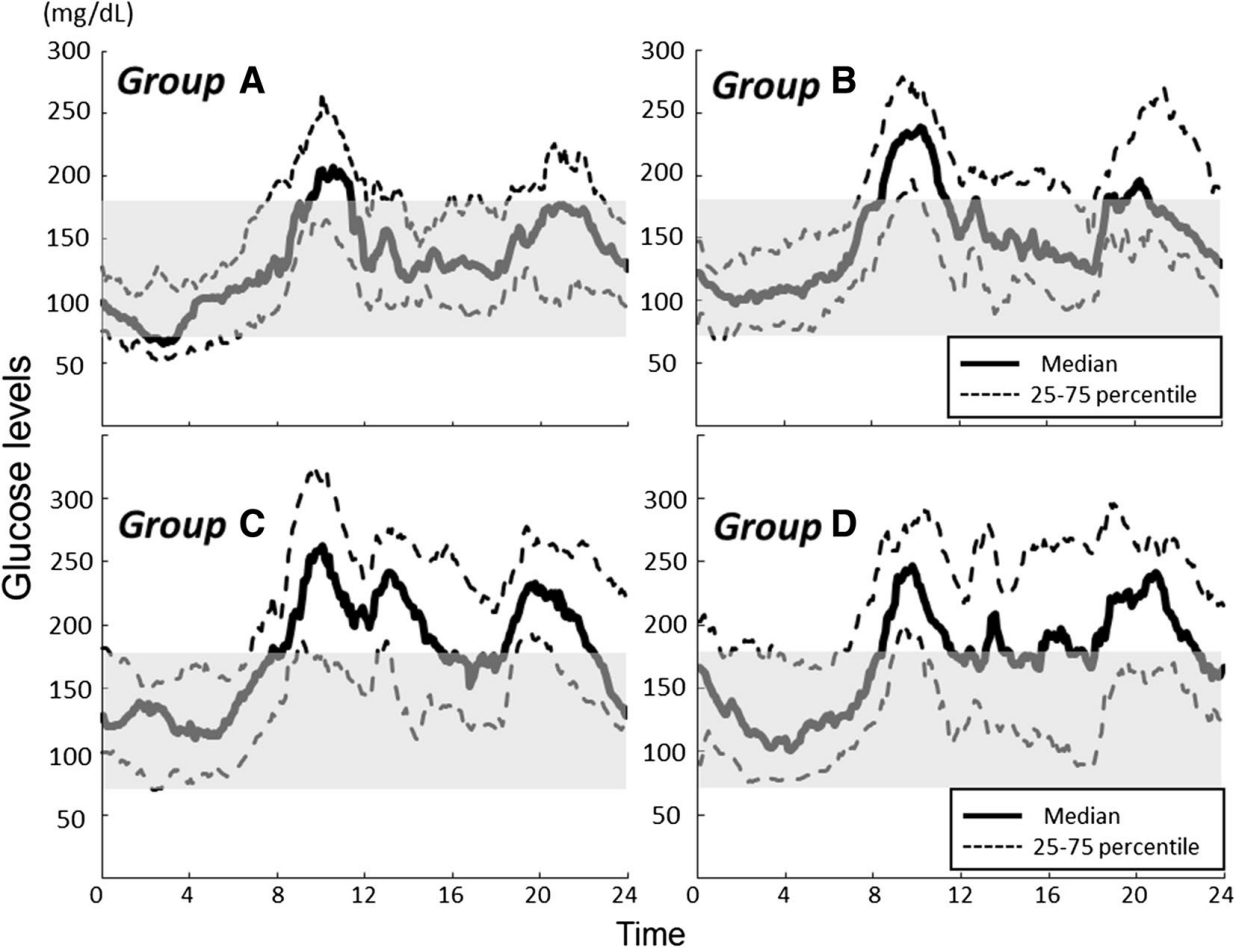
24-h CGM data for 101 type 1 diabetic patients on basal-bolus insulin therapy as stratified equally by HbA1c value. Group A, HbA1c ≤7.2 %; group B, 7.2 % <HbA1c ≤8.2 %; group C, 8.2 % <HbA1c ≤ 9.2 %; and group D, HbA1c >9.2 %

The median glucose values (25–75 percentiles), which were measured every 5 min, were plotted for all patients in each subgroup (Fig. 1), which demonstrated that while the patterns of glycemic variability observed were similar across the subgroups, hypoglycemia occurred mainly during the night in the lower HbA1c subgroups, and hyperglycemia occurred particularly during the day in the higher HbA1c subgroups.

**Fig. 2:**
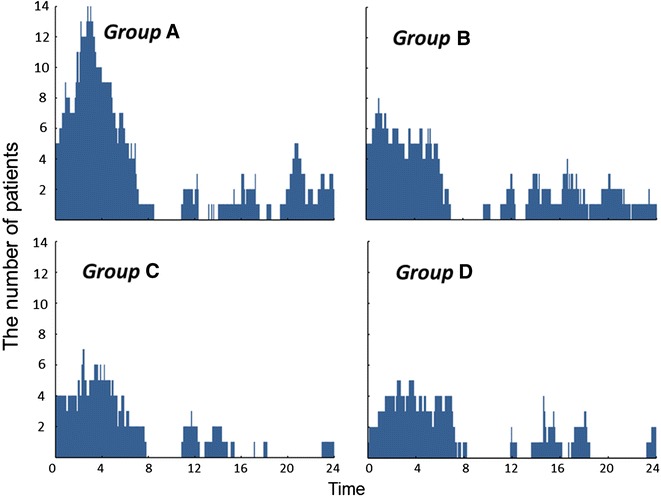
Number of patients experiencing hypoglycemia by time segment in all HbA1c subgroups

In addition, we showed the number of patients experiencing hypoglycemia by time segment in all HbA1c subgroups as a bar graph in Fig. 2, where the patients in all HbA1c subgroups are visually represented as having experienced hypoglycemia most often during the hours from midnight to dawn.

Next, bimodal logistic regression analysis was performed on the presence/absence of hypoglycemia and nocturnal hypoglycemia by using, as independent variables, patient age, BMI, urinary CPR, HbA1c values, TDD, and SDs of 24-h glucose values. These showed that the presence of hypoglycemia was significantly correlated with lower HbA1c values (/%) alone [odds ratio (OR) for hypoglycemia: 0.537; *P* = 0.001], while the presence of nocturnal hypoglycemia was significantly correlated not only with lower HbA1c values (/%) (OR for nocturnal hypoglycemia: 0.590; *P* = 0.003) but also with higher SDs (OR for nocturnal hypoglycemia: 1.026; *P* = 0.045).

## Discussion

It has been shown in the diabetes control and complications trial (DCCT) and its successor, the Epidemiology of Diabetes Interventions and Complications (EDIC) study, that long-term intensive insulin therapy provides reductions not only in microangiopathy but also in macroangiopathy in type 1 diabetic patients [6–10]. In clinical practice, however, despite basal-bolus insulin therapy, quite a few patients with type 1 diabetes are found to exhibit marked glycemic variability, whose whole picture may not be captured by SMBG involving the usual 2–4 measurements a day. Thus, it is highly relevant to capture and provide feedback on patterns of glycemic variability that characterize these patients using masked CGM as a guide to optimizing insulin therapy for them.

In our earlier study evaluating 24-h glycemic variability in 12 type 1 diabetic patients receiving basal-bolus therapy as assessed by masked CGM [11], these patients were shown to have a median HbA1c value of 6.9 % (range, 6.3–8.6), a 24-h median glucose value of 154 mg/dL (range, 108–169), and a median SD of 24-h glucose values of 48 (range, 40–78); with the median range of postprandial glucose increase being 85 mg/dL (range, 46–127) after breakfast, 67 mg/dL (49–111) after lunch, and 75 mg/dL (range, 52–105) after dinner; and the time to postprandial peak glucose values being 100 min (range, 61–134) after breakfast, 65 min (range, 41–109) after lunch, and 78 min (range, 61–149) after dinner. Given that both the range of postprandial glucose increase and the time to postprandial peak glucose values were shown to be greatest after breakfast in these patients, a rationale is suggested for targeting post-breakfast glucose increases for treatment in type 1 diabetic patients.

In the present study, bimodal logistic regression analysis was performed on factors correlated with hypoglycemia and nocturnal hypoglycemia in this study, which revealed that a 1 % increase in HbA1c value is associated with a 46 % decrease in the risk for onset of hypoglycemia as well as a 41 % decrease in the risk for onset of nocturnal hypoglycemia. Also, the higher SDs of 24-h glucose values are correlated with increased risk for the onset of nocturnal hypoglycemia—an extremely important finding on glycemic variability in type 1 diabetes—which suggests that episodes of nocturnal hypoglycemia may adversely affect glycemic fluctuations or, conversely, that excessive glycemic variability may adversely affect episodes of nocturnal hypoglycemia. This is consistent with the results of an earlier study of 81 patients with diabetes using masked CGM [12], which demonstrated that, of the parameters for glycemic variability, the CV of glucose is correlated with the risk for hypoglycemia.

Not being a randomized study, the study has some limitations. There may be confounding factors that may have affected both glycemic variability and mean glucose levels. In Addition, hypoglycemia as assessed during hospital admission may not reflect hypoglycemia as it occurs in daily living.

## Conclusions

101 type 1 diabetic patients receiving basal-bolus insulin therapy were enrolled and their CGM data were collected and subjected to analysis to provide further insight into glycemic variability. When stratified equally by HbA1c values, CGM data demonstrated (i) that all HbA1c subgroups exhibited similar patterns of glycemic variability with the SD of their glucose values being about 50–60 mg/dL in all groups; and (ii) that the lower the HbA1c values, the significantly longer the duration of hypoglycemia and nocturnal (23:00–6:00) hypoglycemia, suggesting that lower HbA1c was not associated with lower SD but may lead to increased hypoglycemic episodes.
